# Postnatal treatment using curcumin supplements to amend the damage in VPA-induced rodent models of autism

**DOI:** 10.1186/s12906-017-1763-7

**Published:** 2017-05-10

**Authors:** Maha Al-Askar, Ramesa Shafi Bhat, Manar Selim, Laila Al-Ayadhi, Afaf El-Ansary

**Affiliations:** 1 Department of Biochemistry, Science College, King Saud University, Riyadh, Saudi Arabia; 2 Department of Zoology, Science College, King Saud University, Riyadh, Saudi Arabia; 3 Department of Physiology, Faculty of Medicine, King Saud University, Riyadh, Saudi Arabia; 4 Central Laboratory, Female Center for Medical Studies and Scientific Section, Riyadh, Saudi Arabia; 5 Autism Research and Treatment Center, Riyadh, Saudi Arabia; 6 Shaik AL-Amodi Autism Research Chair, King Saud University, Riyadh, Saudi Arabia

**Keywords:** Autism, Neurodevelopment valproic acid, Curcumin, Glutathione, Serotonin, Cytokines

## Abstract

**Background:**

Valproic acid (VPA) is used as a first-line antiepileptic agent and is undergoing clinical trials for use as a treatment for many disorders. Mothers undergoing VPA treatment during early pregnancy reportedly show increased rates of autism among their offspring. The benefits of curcumin supplementation were investigated using an animal model of VPA-induced autism.

**Methods:**

The study was performed using a rodent model of autism by exposing rat fetuses to valproic acid (VPA) on the 12.5th day of gestation. At 7 days from their birth, the animals were supplemented with a specific dose of curcumin. Forty neonatal male Western Albino rats were divided into four groups. Rats in group I received only phosphate-buffered saline, rats in group II were the prenatal VPA exposure newborns, rats in group III underwent prenatal VPA exposure supplemented with postnatal curcumin, and rats in group IV were given only postnatal curcumin supplements.

**Results:**

VPA rats exhibited delayed maturation and lower body and brain weights with numerous signs of brain toxicity, such as depletion of IFN-γ, serotonin, glutamine, reduced glutathione, glutathione S-transferase, lipid peroxidase with an increase in CYP450, IL-6, glutamate, and oxidized glutathione. A curcumin supplement moderately corrected these dysfunctions and was especially noticeable in improving delayed maturation and abnormal weight.

**Conclusions:**

Curcumin plays a significant therapeutic role in attenuating brain damage that has been induced by prenatal VPA exposure in rats; however, its therapeutic role as a dietary supplement still must be certified for use in humans.

**Electronic supplementary material:**

The online version of this article (doi:10.1186/s12906-017-1763-7) contains supplementary material, which is available to authorized users.

## Background

Experimental animal models of autism can help researchers understand the etiology of autism in humans and explore various supplements used to amend the impaired biomarkers related to the disease [1]. In reality, autism manifests as a set of behavioral changes, and the behavior of an animal model can never be the same as the behavior of an autistic child, but these behaviors can be scrutinized using precise experiments to measure the behavioral modifications. [2, 3] Currently, different approaches are used to induce human-like autistic features in rodent models by exposing animals to certain chemicals, such as valproic acid (VPA), since VPA significantly increases the rate of autism among the offspring of human mothers who are medicated with VPA during early pregnancy [4]. VPA exposure during the first trimester of conception is associated with risk of autism in the child, particularly if exposure occurs during the time of neural tube closure [5]. Thus, our rodent models showing autistic features were male Wistar neonatal rats originating from valproate-treated females [6]. These females received a single intraperitoneal injection of 600 mg/kg of sodium valproate on day 12.5 after conception.

For many years, VPA, a branched short-chain fatty acid, was used as a first-line antiepileptic agent, particularly in children suffering from epilepsy. [7] Presently, this drug is in clinical trials for use in the treatment of many disorders, however various consequences such as fatal hemorrhaging, pancreatitis, bone marrow suppression, hepatotoxicity, and hyperammonemic encephalopathy are associated with its use. VPA acts on γ amino butyric acid (GABA) levels, changes the activity of many neurotransmitters, and blocks Na + channels, Ca2+ channels and voltage-gated channels in brain tissue [8]. Many studies have shown that valproate exposure in utero is associated with increased risk of neural tube defects, neurodevelopmental deficits and reduced vocal skills. [9–11].

Curcumin is known for its protective actions against various central nervous system disorders such as Alzheimer’s disease, tardive dyskinesia, major depression, epilepsy, neurodegenerative disorders and neuropsychiatric disorders [12]. It can cross the blood brain barrier and is nontoxic at high doses [13]. Many studies have proved that curcumin targets multiple degenerative pathways including oxidative/nitrosative stress, mitochondrial dysfunction, and protein aggregation [14]. Curcumin was effective in ameliorating propionic acid-induced autism in rats through the suppression of oxidative stress, mitochondrial dysfunction and neuroinflammation [14]. All reported biological activities of curcumin could potentially be of interest as autism therapies. Therefore, we studied the therapeutic effects of curcumin in VPA-induced animal models of autism.

## Methods

### Chemicals

Curcumin from *Curcuma longa* (Turmeric) (powder, 50 g in a glass bottle) and valproic acid sodium salt (powder, 25 g in a glass bottle) were obtained from Sigma. The catalog numbers are C1386 and P4543, respectively.

### Animals

Female Wistar rats (180–200 g) that were acclimated in our laboratory under standard laboratory conditions with a controlled fertility cycle were obtained from the Center for Laboratory Animals and Experimental Surgery at King Khalid University Hospital, Riyadh. Rats were mated overnight, and the pregnant rats were divided into 2 sets. On day 12.5 after conception, set I was injected with a single intraperitoneal injection of normal saline, and set II was injected with a single intraperitoneal injection of 600 mg/kg of sodium valproate [15]. Twenty Male Wistar neonatal rats that were born from each set of females (treated with normal saline and VPA) were further divided into two groups of ten pups each. Finally, four groups (10 neonatal rats each) were organized as follows:

Group I (control): Male pups from set I was given an oral dose of 1 ml of normal saline on day 7 after birth.

Group II (VPA rodent model): Male pups from set II (valproate treated mothers) were given an oral dose of 1 ml of normal saline on day 7 after birth.

Group III (VPA-Curcumin): Male pups from set II (valproate treated mothers) received 1 ml of curcumin (1 g/kg b.wt) [16] orally at 7 days after birth.

**Fig. 1:**
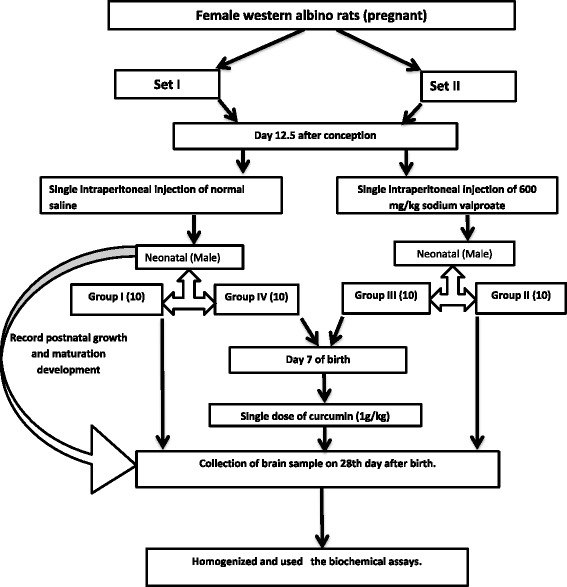
Schematic presentation of the designed experiment

Group IV (Curcumin): Male pups from set I received 1 ml of curcumin (1 g/kg b.wt) [16] orally at 7 days after birth. Figure 1 summarizes the experimental design and the different groups that were studied.

#### Postnatal growth and maturation development

Animals were weighed at 0, 7, 14, 21 and 27 days after delivery. See the uploaded diagram that illustrates the experimental design.

### Tissue preparation

On the 28th day after birth, all groups were killed by decapitation. The brains were quickly collected, weighed, washed with normal saline and then homogenized in 10 times *w*/*v* bi-distilled water and further used in the biochemical assays.

### Biochemical analyses

Tissue samples were run concordant with the instructions of the kit protocol. The quantification of interleukin-6, interferon gamma, and reduced glutathione in the brain tissue were determined using a rat ELISA Kit obtained from “My Bio Source” that were based on a quantitative sandwich immunoassay technique, while for cytochrome P450, enzyme-linked immune sorbent assay, based on the biotin double antibody sandwich technology obtained from “My Bio Source”, was used. The quantification of lipid peroxide, glutathione S-transferases, glutamine, and glutamate in the brain tissue was determined using the ELISA Kit based on a quantitative sandwich immunoassay technique obtained from “Cusabioin”.

The quantification of serotonin in the brain tissue was carried out using a 5-HT ELISA Kit, which applies the competitive enzyme immunoassay technique utilizing a monoclonal anti-5-HT antibody and a 5HT-HRP conjugate, and for oxidized glutathione, a GSSG ELISA kit was used, which applies the competitive enzyme immunoassay technique utilizing a monoclonal anti-GSSG antibody and a GSSG-HRP conjugate, which were obtained from My Bio Source.

### Statistical analysis

The Statistical Package for the Social Sciences (SPSS) computer program was used. The results were expressed as the mean ± S.D., and all statistical comparisons were made using independent T-Tests, with values of *P* ≤ 0.05 considered to be significant. Pearson’s correlations were also performed, and the best fit line was drawn. Receiver operating characteristics (ROC) analysis was performed. Area under the curve, cutoff values threshold, and degrees of specificity and sensitivity were calculated.

## Results

The analysis of the body weight, brain weight and eye opening age in pups showed statistically significant (*P* < 0.001) differences in all tested groups compared with the control group, as shown in Table 1. VPA-exposed rats showed delayed maturation, as represented by lower body weight, a slight decrease in brain weight and late eye opening compared to the control group, whereas curcumin treatment was effective in promoting body and brain weight in VPA-exposed pups (Table 1).

**Table 1:** Mean ± S.D. together with the independent t-test for weight gain, brain weight and age of eye opening between neurointoxicated, protected and therapeutically treated rat pups compared to healthy control

Parameter	Group	N	Min	Max	Mean ± S.D.	Percent Change	*P* value^a^	*P* value^b^
Started weight (g)	Control	10	14.80	20.00	17.62 ± 1.96	100.00		
	VPA	10	14.90	18.80	17.47 ± 1.38	99.15	0.845	0.001
	VPA-CUR	10	24.50	31.70	27.81 ± 2.82^c^	157.83	0.001	
	CUR	10	15.20	18.20	16.46 ± 1.04	93.42	0.121	
Final weight (g)	Control	10	26.10	78.90	58.42 ± 21.45	100.00		0.001
	VPA	10	17.20	75.40	42.21 ± 27.85	72.25	0.163	
	VPA-CUR	10	69.30	100.30	88.18 ± 12.10^c^	150.94	0.002	
	CUR	10	55.60	70.00	63.22 ± 5.61	108.22	0.509	
Weight Gained	Control	10	11.30	58.90	40.80 ± 19.56	100.00		0.001
	VPA	10	-0.30	60.30	24.74 ± 28.56	60.64	0.162	
	VPA-CUR	10	44.80	72.90	60.37 ± 9.80^c^	147.97	0.014	
	CUR	10	40.40	51.80	46.76 ± 4.80	114.61	0.371	
Brain weight (g)	Control	10	1.17	1.61	1.38 ± 0.15	100.00		
	VPA	10	1.12	1.63	1.31 ± 0.17	95.08	0.361	0.001
	VPA-CUR	10	1.51	1.72	1.61 ± 0.06^c^	116.54	0.001	
	CUR	10	1.29	1.59	1.43 ± 0.09	103.58	0.377	
Opening eyes (Days)	Control	10	15.00	16.00	15.30 ± 0.48	100.00		0.001
	VPA	10	10.00	17.00	14.50 ± 3.21	94.77	0.454	
	VPA-CUR	10	14.00	16.00	15.20 ± 1.03	99.35	0.786	
	CUR	10	11.00	13.00	12.0 0.47^c^	78.43	0.001	

^a^ *P* value between control group and other groups

^b^ *P* value between all groups

^c^Indicates there is significant difference between the group and control at 0.05 level.

Table 2 exhibits the significant depletion of IFN-γ and non-significant depletion of 5HT and glutamine upon VPA exposure compared to the control group. CYP450 was significantly increased, while IL-6 and glutamate were non-significantly increased in VPA-exposed pups, and curcumin was effective in restoring nearly all the parameters, as shown in Table 2.

**Table 2:** Mean ± S.D. together with the independent t-test for Interleukin-6, Interferon Gamma, cytochrome P450, Serotonin, Glutamine, Glutamate together with Glutamate/Glutamine Ratio in neuro-intoxicated, protected and therapeutically treated rat pups compared to healthy control

Parameter	Group	N	Min	Max	Mean ± S.D.	Percent Change	*P* value^a^	*P* value^b^
IL-6 (pg\ml)	Control	10	4.50	22.29	12.59 ± 6.07	100.00		0.019
	VPA	10	3.61	24.96	15.47 ± 7.56	122.86	0.360	
	VPA-CUR	10	4.50	11.61	7.96 ± 2.22	63.25	0.044	
	CUR	10	3.61	16.95	9.87 ± 3.86	78.36	0.246	
IFN-γ (pg\ml)	Control	10	206.47	407.59	331.64 ± 87.78	100.00		0.038
	VPA	10	152.14	343.25	249.08 ± 58.88	75.10	0.024	
	VPA-CUR	10	167.86	366.84	244.41 ± 69.94^c^	73.70	0.024	
	CUR	10	117.82	359.21	237.26 ± 95.02^c^	71.54	0.033	
CYP450 (ng\ml)	Control	10	33.02	40.16	36.46 ± 1.79	100.00		0.085
	VPA	10	33.85	42.01	39.11 ± 2.64	107.27	0.017	
	VPA-CUR	10	34.62	43.83	37.50 ± 2.82	102.87	0.335	
	CUR	10	34.14	43.83	38.96 ± 2.92	106.85	0.033	
5HT (ng\ml)	Control	10	113.92	169.46	148.75 ± 15.22	100.00		0.228
	VPA	10	109.57	174.05	140.14 ± 22.04	94.22	0.323	
	VPA-CUR	10	85.18	181.05	135.77 ± 32.81	91.28	0.278	
	CUR	10	122.13	215.83	158.84 ± 30.84	106.78	0.366	
Glutamine (pmol\ml)	Control	10	1640.31	2540.39	2186.87 ± 302.70	100.00		0.001
	VPA	10	1861.71	2549.07	2082.25 ± 216.82	95.22	0.386	
	VPA-CUR	10	1456.53	2052.73	1763.15 ± 165.27^c^	80.62	0.002	
	CUR	10	1388.51	2324.78	1798.91 ± 274.83^c^	82.26	0.008	
Glutamate (nmol\ml)	Control	10	215.20	286.01	249.53 ± 24.68	100.00		0.002
	VPA	10	235.78	283.18	258.51 ± 14.04	103.60	0.334	
	VPA-CUR	10	178.70	283.58	232.05 ± 34.97	92.99	0.213	
	CUR	10	168.81	272.09	206.71 ± 39.12^c^	82.84	0.009	
Glutamate/Glutamine Ratio	Control	10	0.10	0.16	0.116 ± 0.018	100.00		0.063
	VPA	10	0.11	0.14	0.125 ± 0.011	108.04	0.169	
	VPA-CUR	10	0.10	0.16	0.132 ± 0.017	113.83	0.051	
	CUR	10	0.10	0.14	0.115 ± 0.016	99.65	0.963	

^a^ *P* value between control group and other groups

^b^ *P* value between all groups

^c^Indicates there is significant difference between the group and control at 0.05 level

**Fig. 2:**
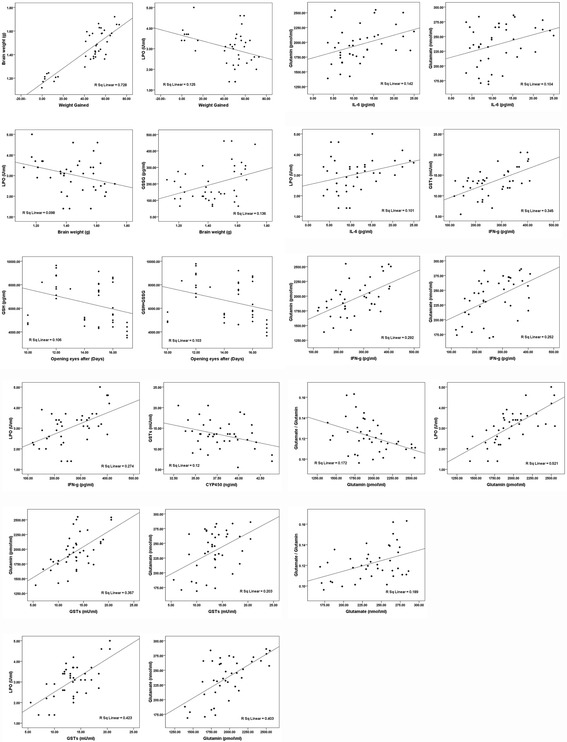
Correlation between all parameters with best fit line curve

Table 3 shows lipid peroxide, oxidized glutathione, reduced glutathione, and glutathione S-transferases levels in all of the treated groups, along with the GSH/GSSG ratios. Non-significant decreases in LPO and GSTs in the VPA group were observed in all treated groups compared to the control group. The same table demonstrates the significant decrease in GSH in VPA and VPA-CUR compared to the control group. GSSG showed a significant increase in all of the treated pups compared with the control group. Table 4 and Fig. 2 present the Pearson’s correlations between the measured parameters.

**Table 3:** Mean ± S.D. together with the independent t-test for Lipid Peroxide, Oxidized Glutathione, Reduced Glutathione, Glutathione S-transferase together with GSH/GSSG ratio between neuro -intoxicated, protected and therapeutically treated rat pups compared to healthy control

Parameter	Group	N	Min	Max	Mean ± S.D.	Percent Change	*P* value^a^	*P* value^b^
LPO (U\ml)	Control	10	2.70	5.00	3.68 ± 0.84	100.00		0.001
	VPA	10	3.10	3.90	3.52 ± 0.24	95.65	0.573	
	VPA-CUR	10	1.40	3.40	2.46 ± 0.55^c^	66.73	0.001	
	CUR	10	1.40	3.70	2.56 ± 0.82^c^	69.57	0.007	
GSSG (pg/ml)	Control	10	65.00	125.00	92.50 ± 23.24	100.00		0.001
	VPA	10	150.00	310.00	225.00 ± 51.91^c^	243.24	0.001	
	VPA-CUR	10	225.00	460.00	345.00 ± 81.99^c^	372.97	0.001	
	CUR	10	100.00	180.00	138.50 ± 21.48	149.73	0.001	
GSH (pg\ml)	Control	10	7104.06	9134.06	8016.97 ± 638.64	100.00		0.001
	VPA	10	3506.25	5417.81	4397.66 ± 557.53^c^	54.85	0.001	
	VPA-CUR	10	4419.06	6040.94	5099.31 ± 485.93^c^	63.61	0.001	
	CUR	10	6825.00	9638.75	8190.53 ± 948.88	102.16	0.637	
GSTs (mU\ml)	Control	10	8.50	20.50	15.35 ± 3.98	100.00		0.001
	VPA	10	11.60	18.40	13.84 ± 2.00	90.19	0.304	
	VPA-CUR	10	8.50	17.00	12.74 ± 2.74	83.00	0.105	
	CUR	10	5.50	17.00	11.57 ± 3.78^c^	75.37	0.043	
GSH/GSSG	Control	10	59.15	140.52	92.59 ± 27.69	100.00		0.001
	VPA	10	13.87	31.68	20.54 ± 5.53^c^	22.19	0.001	
	VPA-CUR	10	9.61	22.22	15.64 ± 4.24^c^	16.89	0.001	
	CUR	10	47.43	87.58	60.33 ± 11.63^c^	65.16	0.005	

^a^ *P* value between control group and other groups

^b^ *P* value between all groups

^c^Indicates there is significant difference between the group and control at 0.05 level

**Table 4:** Pearson Correlations between the measured parameters

Parameters	R (Person Correlation)	Sig.	
Started weight (g) ~ Final weight (g)	0.641^**^	0.001	P^a^
Started weight (g) ~ Weight Gained	0.494^**^	0.001	P^a^
Started weight (g) ~ Brain weight (g)	0.652^**^	0.001	P^a^
Started weight (g) ~ Opening eyes after (Days)	0.313^*^	0.049	P^a^
Started weight (g) ~ Glutamate/Glutamin Ratio	0.322^*^	0.043	P^a^
Started weight (g) ~ GSH (mmol/L)	−0.438^**^	0.005	N^b^
Started weight (g) ~ GSSG (pg/ml)	0.765^**^	0.001	P^a^
Started weight (g) ~ GSH (pg\ml)	−0.438^**^	0.005	N^b^
Started weight (g) ~ GSH + GSSG	-0.408^**^	0.009	N^b^
Started weight (g) ~ GSH/GSSG	-0.479^**^	0.002	N^b^
Final weight (g) ~ Weight Gained	0.984^**^	0.001	P^a^
Final weight (g) ~ Brain weight (g)	0.887^**^	0.001	P^a^
Final weight (g) ~ LPO (U\ml)	−0.371^*^	0.019	N^b^
Final weight (g) ~ GSSG (pg/ml)	0.390^*^	0.013	P^a^
Weight Gained ~ Brain weight (g)	0.853^**^	0.001	P^a^
Weight Gained ~ LPO (U\ml)	−0.353^*^	0.025	N^b^
Brain weight (g) ~ LPO (U\ml)	−0.313^*^	0.049	N^b^
Brain weight (g) ~ GSSG (pg/ml)	0.369^*^	0.019	P^a^
Opening eyes after (Days) ~ GSH (mmol/L)	−0.326^*^	0.04	N^b^
Opening eyes after (Days) ~ GSH (pg\ml)	−0.326^*^	0.04	N^b^
Opening eyes after (Days) ~ GSH + GSSG	-0.322^*^	0.043	N^b^
IL-6 (pg\ml) ~ Glutamin (pmol\ml)	0.377^*^	0.017	P^a^
IL-6 (pg\ml) ~ Glutamate (nmol\ml)	0.322^*^	0.043	P^a^
IL-6 (pg\ml) ~ LPO (U\ml)	0.318^*^	0.045	P^a^
IFN-g (pg\ml) ~ GSTs (mU\ml)	0.588^**^	0.001	P^a^
IFN-g (pg\ml) ~ Glutamin (pmol\ml)	0.541^**^	0.001	P^a^
IFN-g (pg\ml) ~ Glutamate (nmol\ml)	0.502^**^	0.001	P^a^
IFN-g (pg\ml) ~ LPO (U\ml)	0.523^**^	0.001	P^a^
CYP450 (ng\ml) ~ GSTs (mU\ml)	−0.347^*^	0.028	N^b^
5HT (ng\ml) ~ GSSG (pg/ml)	−0.352^*^	0.026	N^b^
GSTs (mU\ml) ~ Glutamin (pmol\ml)	0.597^**^	0.001	P^a^
GSTs (mU\ml) ~ Glutamate (nmol\ml)	0.451^**^	0.004	P^a^
GSTs (mU\ml) ~ LPO (U\ml)	0.650^**^	0.001	P^a^
Glutamin (pmol\ml) ~ Glutamate (nmol\ml)	0.635^**^	0.001	P^a^
Glutamin (pmol\ml) ~ Glutamate/Glutamin Ratio	−0.414^**^	0.008	N^b^
Glutamin (pmol\ml) ~ LPO (U\ml)	0.722^**^	0.001	P^a^
Glutamate (nmol\ml) ~ Glutamate/Glutamin Ratio	0.435^**^	0.005	P^a^
Glutamate (nmol\ml) ~ LPO (U\ml)	0.678^**^	0.001	P^a^
Glutamate/Glutamin Ratio ~ GSSG (pg/ml)	0.337^*^	0.033	P^a^
GSH (mmol/L) ~ GSSG (pg/ml)	−0.684^**^	0.001	N^b^
GSH (mmol/L) ~ GSH (pg\ml)	1.000^**^	0.001	P^a^
GSH (mmol/L) ~ GSH + GSSG	0.999^**^	0.001	P^a^
GSH (mmol/L) ~ GSH/GSSG	0.819^**^	0.001	P^a^
GSSG (pg/ml) ~ GSH (pg\ml)	−0.684^**^	0.001	N^b^
GSSG (pg/ml) ~ GSH + GSSG	-0.651^**^	0.001	N^b^
GSSG (pg/ml) ~ GSH/GSSG	-0.816^**^	0.001	N^b^
GSH (pg\ml) ~ GSH + GSSG	0.999^**^	0.001	P^a^
GSH (pg\ml) ~ GSH/GSSG	0.819^**^	0.001	P^a^
GSH + GSSG ~ GSH/GSSG	0.803^**^	0.001	P^a^

**Correlation is significant at the 0.01 level.

*Correlation is significant at the 0.05 level.

^a^Positive Correlation.

^b^Negative Correlation

**Fig. 3:**
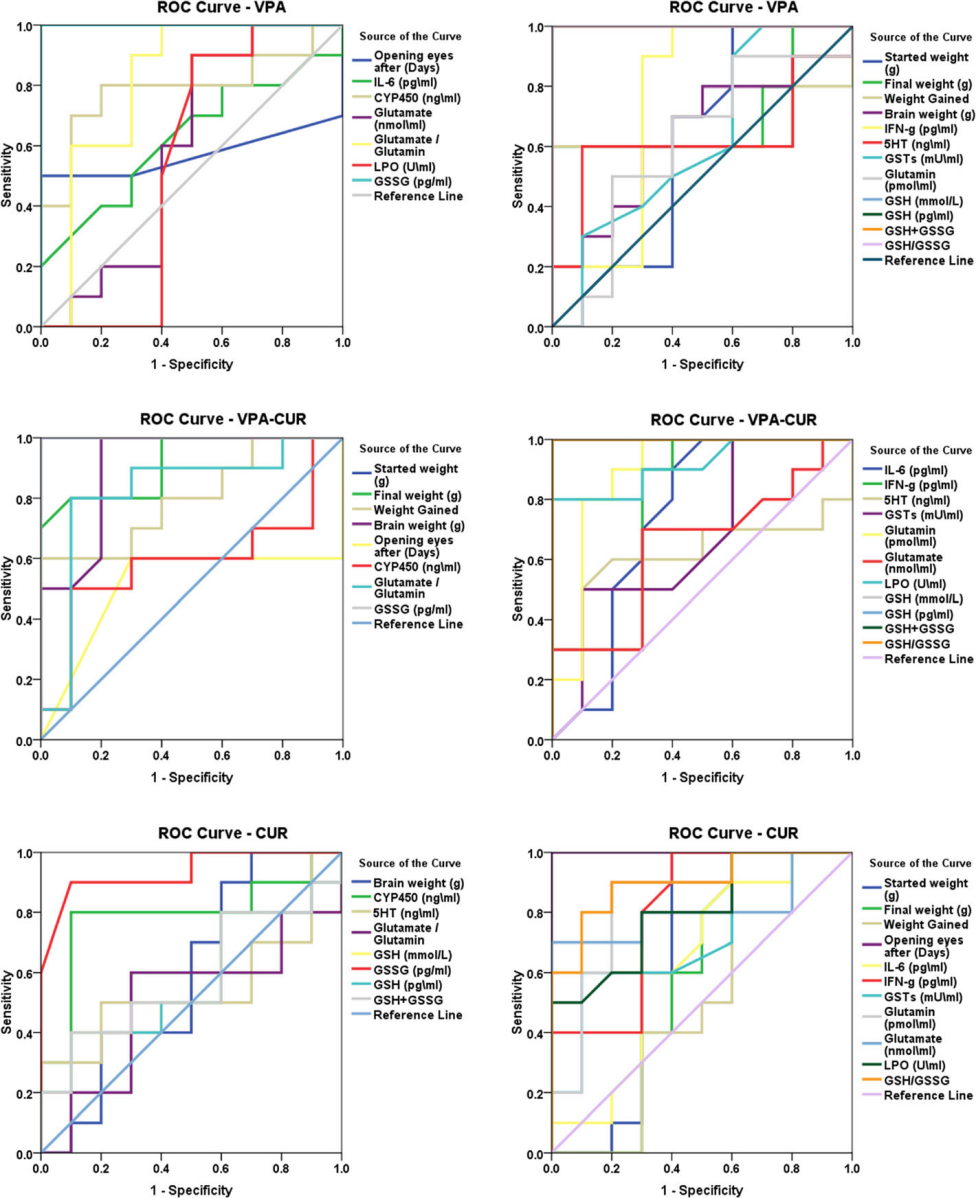
ROC Curve of all parameters for all groups

Receiver operating characteristics curves are presented in Fig. 3. Area under the curve (AUC), cutoff values, sensitivity and specificity are listed in Tables 5, 6 and 7

**Table 5:** ROC Curve of weight gain, brain weight and age of eye opening between neuro-intoxicated, protected and therapeutically treated rat pups compared to healthy control

Parameters	Groups	Area under the curve	Cutoff value	Sensitivity %	Specificity %
Started weight (g)	VPA	0.600	18.900	100.0%	40.0%
	VPA-CUR	1.000	22.250	100.0%	100.0%
	CUR	0.670	18.250	100.0%	60.0%
Final weight (g)	VPA	0.700	25.450	60.0%	100.0%
	VPA-CUR	0.915	77.550	80.0%	90.0%
	CUR	0.585	71.300	100.0%	40.0%
Weight Gained	VPA	0.640	8.700	60.0%	100.0%
	VPA-CUR	0.800	60.200	60.0%	100.0%
	CUR	0.530	52.700	100.0%	40.0%
Brain weight (g)	VPA	0.640	1.415	80.0%	50.0%
	VPA-CUR	0.905	1.477	100.0%	80.0%
	CUR	0.590	1.364	90.0%	40.0%
Opening eyes after (Days)	VPA	0.570	16.500	50.0%	100.0%
	VPA-CUR	0.510	15.500	60.0%	70.0%
	CUR	1.000	14.000	100.0%	100.0%

**Table 6:** ROC Curve of Interleukin-6, Interferon Gamma, cytochrome P450, Serotonin, Glutamine, Glutamate together with Glutamate/Glutamine ratio in neuro-intoxicated, protected and therapeutically treated rat pups compared to healthy control

Parameters	Groups	Area under the curve	Cutoff value	Sensitivity %	Specificity %
IL-6 (pg\ml)	VPA	0.625	9.832	80.0%	40.0%
	VPA-CUR	0.740	11.165	90.0%	60.0%
	CUR	0.635	12.945	80.0%	50.0%
IFN-γ (pg\ml)	VPA	0.750	318.225	90.0%	70.0%
	VPA-CUR	0.780	327.520	90.0%	70.0%
	CUR	0.805	365.885	100.0%	60.0%
CYP450 (ng\ml)	VPA	0.790	37.085	80.0%	80.0%
	VPA-CUR	0.590	37.471	50.0%	90.0%
	CUR	0.790	37.630	80.0%	90.0%
5HT (ng\ml)	VPA	0.620	137.220	60.0%	90.0%
	VPA-CUR	0.625	140.965	60.0%	80.0%
	CUR	0.550	157.750	50.0%	80.0%
GSTs (mU\ml)	VPA	0.620	17.100	90.0%	40.0%
	VPA-CUR	0.675	12.450	50.0%	90.0%
	CUR	0.730	12.450	60.0%	90.0%
Glutamin (pmol\ml)	VPA	0.610	2365.295	90.0%	40.0%
	VPA-CUR	0.890	1984.715	90.0%	80.0%
	CUR	0.840	2007.865	90.0%	80.0%
Glutamate (nmol\ml)	VPA	0.590	244.955	90.0%	50.0%
	VPA-CUR	0.645	234.870	70.0%	70.0%
	CUR	0.810	213.790	70.0%	100.0%
Glutamate/Glutamin ratio	VPA	0.810	0.111	100.0%	60.0%
	VPA-CUR	0.820	0.122	80.0%	90.0%
	CUR	0.500	0.115	60.0%	70.0%

**Table 7:** ROC Curve of Lipid Peroxide, Oxidized Glutathione, Reduced Glutathione, Glutathione S-transferase together with GSH/GSSG ratio between neuro-intoxicated, protected and therapeutically treated rat pups compared to healthy control

Parameters	Groups	Area under the curve	Cutoff value	Sensitivity %	Specificity %
LPO (U\ml)	VPA	0.545	3.250	90.0%	50.0%
	VPA-CUR	0.915	2.650	80.0%	100.0%
	CUR	0.805	3.150	80.0%	70.0%
GSH (mmol/L)	VPA	1.000	200.350	100.0%	100.0%
	VPA-CUR	1.000	210.320	100.0%	100.0%
	CUR	0.570	278.195	40.0%	90.0%
GSSG (pg/ml)	VPA	1.000	137.500	100.0%	100.0%
	VPA-CUR	1.000	175.000	100.0%	100.0%
	CUR	0.935	117.500	90.0%	90.0%
GSH (pg\ml)	VPA	1.000	6260.938	100.0%	100.0%
	VPA-CUR	1.000	6572.500	100.0%	100.0%
	CUR	0.570	8693.594	40.0%	90.0%
GSH + GSSG	VPA	1.000	6435.938	100.0%	100.0%
	VPA-CUR	1.000	6762.500	100.0%	100.0%
	CUR	0.580	8798.594	40.0%	90.0%
GSH/GSSG	VPA	1.000	45.417	100.0%	100.0%
	VPA-CUR	1.000	40.685	100.0%	100.0%
	CUR	0.900	71.255	90.0%	80.0%

## Discussion

Prenatal VPA exposure resulted in delayed maturation in newborns, as evidenced by lower body weight, a slight decrease in brain weight and late eye opening, indicating some altered neurodevelopmental effects. Our results validate many previous studies that suggest there is a maturational delay in the early stage of life of VPA-exposed rats [17]. Significant effects of postnatal curcumin treatments were found, that ameliorated all of the observed development delays in the current study. These results indicate the therapeutic potential of curcumin as a neuroprotective agent in the treatment of delayed maturation. Our results are consistent with many recent studies on discovering redevelopment and accelerating motor functional recovery of curcumin treatment in mice [18].

Elevated levels of IL-6 in the CNS have been reported in a number of neurological diseases that are associated with brain injury or inflammation [19]. In the current study, VPA exposure amplified the level of IL-6 in the brain tissue, which may be due to neuroinflammation and altering the immune response during brain development. However, curcumin treatment was able to decrease IL-6 levels, as curcumin can suppress the pro-inflammatory gene expression by blocking phosphorylation of the inhibitory factor I-kappa B (IκB) [20]. VPA is generally used in the treatment of epilepsy, but recently, it has been found to be effective in the treatment of oncolytic herpes simplex virus (oHSV) infection, as this drug can inhibit the expression of IFN-β and the IFN-mediated proteins STAT1 and PKR in infected cells [21]. This could support our results showing a significant decrease of IFN-ɣ in VPA-exposed pups. Additionally, the remarkable decrease in this parameter in curcumin-treated pups may be due to the anti-inflammatory action of curcumin, which causes inhibition of production of cytokines, such as interferon-ɣ, due to suppression of the Janus kinase (JAK)-STAT signaling cascade [22]. Cytochrome P450 was found to be increased in VPA-exposed pups while a slight decrease was observed in VPA-CUR and CUR groups, as shown in Table 2. The cytochrome P450 enzymes are the major catalysts involved in the metabolism of many psychoactive drugs in the brain. The significant increase in CYT-P450 in VPA-exposed pups could be due to the availability of VPA as a substrate. Curcumin, a good antioxidant, was slightly effective in decreasing the activity of CYT-P450 [23]. The role of CYT-P450 in the metabolism of VPA can also be explained through non-significant changes in the concentration of lipid peroxides as markers of oxidative stress, as demonstrated in Table 3. The same table demonstrates the antioxidant effects of curcumin, as shown by significant decreases of lipid peroxides in VPA-CUR and CUR groups compared to the VPA group. An unexpected finding of the present study is that VPA does not induce elevation of lipid peroxides as markers of oxidative stress. This finding could be attributed to the fact that VPA is an anti-epileptic drug that is designed to have the least toxic effects on treated patients. The significant decrease in lipid peroxide levels in VPA-CUR and CUR groups compared to the control group is consistent with various models posed by several authors, which proves that curcumin is a good antioxidant that inhibits lipid peroxidation [24].

Glutathione-S-transferases (GSTs) play a key role in enzymatic detoxification and were found to be 10.19% lower in the VPA group than in the control group (Table 3), which is due to the neurotoxic effect of VPA through insufficient conjugation of electrophiles and detoxification of the reactive species, as previously described by Chaudhary & Parvez, in the cerebellum and cerebral cortex of the rat brain [25]. Rats fed dietary curcumin were found to have decreased hepatic GST activity compared to controls because of competitive enzyme inhibition by the curcumin molecule [26]. This could explain the remarkable decrease of GST in the VPA-CUR and CUR groups.

Table 2 shows the non-significant decreased level of serotonin in the VPA group compared to the control group. On the other hand, an increase in serotonin in the CUR group was observed compared to the control, the VPA and the VPA-CUR groups, with values of 6.78, 12.56 and 15.5%, respectively. This is not consistent with a previous study, in which increased levels of serotonin were found in the brains of rats that had been prenatally exposed to VPA in association with disrupted sleep/awake rhythms [27]. Aside from the well-known deficiency in serotonergic neurotransmission as a pathophysiological correlate of autism, recent evidence points to the pivotal role of increased glutamate receptor activation as well. While the present study demonstrates a non-significant decrease in brain serotonin of VPA-exposed rats, a remarkable elevation in brain glutamate was recorded. A hypothesis integrating current concepts of neurotransmission and hypothalamus-pituitary-adrenal (HPA) axis dysregulation with findings on immunological alterations was proposed by Müller and Schwarz [28]. Immune activation, including increased production of pro-inflammatory cytokines, has repeatedly been described in mild depression. Pro-inflammatory cytokines such as IL-2 and IL-6 activate the tryptophan- and serotonin-degrading enzyme indole amine 2,3-dioxygenase (IDO). Based on this hypothesis, the increase in IL-6 reported in the present study can be related to the decrease in serotonin levels. A VPA-exposed developmental rodent model in the present study may show persistent autistic features that present biochemically as low serotonin and high glutamate levels [29].

Glutamate is an excitatory neurotransmitter that is usually transported from neurons to astrocytes in order to be buffered through the formation of glutamine; the glutamate/glutamine ratio can be a useful marker for the decrease of excitotoxicity. Table 3 shows the non-significant elevation of glutamate along with the unchanged glutamine and glutamate/glutamine ratio in the VPA-exposed rats compared to the control group. This is supported by the previous work of Bristot Silvestrin et al. [30], which reported unaltered glutamate uptake in 15 day old rats that were prenatally exposed to VPA and showed a 160% increase at an age of 120 days. The anti-excitotoxicity effect of curcumin, presented as a much lower glutamate level in cur-treated rats compared to VPA-exposed rats, and even the control group, can be easily related to its protective effect against glutamate excitotoxicity [31].

GSH was significantly lower in VPA-exposed rat pups (7 days old), compared to the control group (Table 3) This is consistent with the recent study by Bristot Silvestrin et al. [30], which recorded unaltered GSH levels in 15-day-old rats that were prenatally exposed to VPA. This can be related to the non-significant elevation in glutamate reported in the present study. The unchanged GSH and glutamate levels recorded in the present study do not contradict the use of VPA-exposed rats as rodent models of autism. This opinion can be supported with the previously mentioned significant impairment of both parameters in 120-day-old rats that were exposed to VPA during pregnancy. The neurotoxic effect of VPA, along with the neuro-therapeutic and antioxidant effects of curcumin can be observed together in Table 3.9. A highly significant decrease in GSSG in VPA-exposed rats reflects the impairment of total antioxidant and glutathione status in this group of animals. GSSG, as an oxidized form of glutathione, can be easily converted to GSH by glutathione reductase, and hence, a lower concentration can easily lead to low GSH. In our study, VPA-exposed rats were under stressful conditions, so the unexpected increase of GSSG in valproate-treated animals that were also treated with curcumin, is supported by the previous study by Hagl et al. [32], who reported that when under non-stressful conditions, curcumin induces the synthesis of GSH and many detoxifying enzymes (as shown in group IV). This might also be attributed to the low absorption and quick elimination of curcumin from the body. Hagl et al. proved that low bioavailability of curcumin can be ameliorated through administration with secondary plant compounds, micronization and micellation, which might help to increase its therapeutic potency [32].

Tables 5, 6 and 7 present the ROC curve parameters of all measured variables from all test groups. It is readily apparent that while some parameters show effectiveness as biomarkers for VPA neurotoxicity, others are good to excellent markers for CUR therapeutic and/or antioxidant effects. The postnatal growth and maturation markers presented by weight gain, brain weight and eye opening demonstrated AUC ranges between 0.7 and 1, which support their use as markers for VPA neurotoxicity and curcumin therapeutic potency. IL-6 shows smaller AUC values compared to IFN-γ, which suggests that the latter is a good marker for VPA toxicity and curcumin therapeutic and antioxidant effects. Lipid peroxides and CYT P450 both demonstrate a satisfactory AUC, which shows that both can be used to test the efficacy of the curcumin antioxidant effect. Serotonin, GST, glutamate and glutamine all showed relatively poor potency as markers for VPA neurotoxicity and were good markers for curcumin efficacy. Conversely, GSSG and GSH both show a high predictive value that can be used as markers of VPA neurotoxicity and of curcumin’s therapeutic effect. The increase in GSSG in valproate-treated animals that were also treated with curcumin was unexpected but is supported in the previous study by Reyes et al. (2013), who reported that under non-stress conditions, curcumin induces the synthesis of GSH and of many detoxifying and cytoprotective enzymes. Based on this, our data suggest that pretreatment with curcumin may have more protective effects than therapeutic antioxidant effects but still demonstrates therapeutic effects in ameliorating IL-6, INF-ɣ, LOP, GST,5-HT and GSH.

## Conclusion

To summarize, this study shows evidence of the postnatal therapeutic role of curcumin in improving most of the impaired parameters in VPA-induced rodent models with persistent autistic features. The mechanism of action underlying the therapeutic effects of curcumin should be investigated in the near future. Studies of the protective effects of curcumin are also recommended.

## Additional file

Additional file 1: CAM curcumine data. (XLSX 50 kb)
